# Evaluation of Hand Action Classification Performance Using Machine Learning Based on Signals from Two sEMG Electrodes

**DOI:** 10.3390/s24082383

**Published:** 2024-04-09

**Authors:** Hope O. Shaw, Kirstie M. Devin, Jinghua Tang, Liudi Jiang

**Affiliations:** School of Engineering, Faculty of Engineering and Physical Sciences, University of Southampton, Southampton SO17 1BJ, UK; k.devin@soton.ac.uk (K.M.D.);

**Keywords:** sEMG, hand actions, machine learning, classification, upper limb, myoelectric prosthetics

## Abstract

Classification-based myoelectric control has attracted significant interest in recent years, leading to prosthetic hands with advanced functionality, such as multi-grip hands. Thus far, high classification accuracies have been achieved by increasing the number of surface electromyography (sEMG) electrodes or adding other sensing mechanisms. While many prescribed myoelectric hands still adopt two-electrode sEMG systems, detailed studies on signal processing and classification performance are still lacking. In this study, nine able-bodied participants were recruited to perform six typical hand actions, from which sEMG signals from two electrodes were acquired using a Delsys Trigno Research+ acquisition system. Signal processing and machine learning algorithms, specifically, linear discriminant analysis (LDA), k-nearest neighbors (KNN), and support vector machines (SVM), were used to study classification accuracies. Overall classification accuracy of 93 ± 2%, action-specific accuracy of 97 ± 2%, and F1-score of 87 ± 7% were achieved, which are comparable with those reported from multi-electrode systems. The highest accuracies were achieved using SVM algorithm compared to LDA and KNN algorithms. A logarithmic relationship between classification accuracy and number of features was revealed, which plateaued at five features. These comprehensive findings may potentially contribute to signal processing and machine learning strategies for commonly prescribed myoelectric hand systems with two sEMG electrodes to further improve functionality.

## 1. Introduction

Myoelectric prosthetic hands, controlled by surface electromyography (sEMG) signals generated from contractions of muscles (for example, flexor and extensor carpi radialis) within a residual limb are commonly used to restore hand functions for those with upper limb deficiencies, such as upper limb amputees. To date, many prescribed myoelectric hands still adopt two-electrode sEMG designs [1,2,3]. Comprehensive training is often provided to upper limb amputees in clinics to help amputees adapt to myoelectric prosthetic hand use when conducting activities of daily living. Training often involves use of clinically friendly tools such as the MyoBoy system (Ottobock, Duderstadt, Germany) [4], which is also a two-electrode sEMG system. For a myoelectric prosthetic hand system with two sEMG electrodes, the two electrodes are usually located on the lateral and medial side of the residual limb. Corresponding sEMG signals generated during muscle contractions are intrinsically processed to control prosthetic hand actions, such as open and close functions for single-grip hands [5].

Although single-grip hands are widely prescribed, high rejection rates (of up to 44%) are still common [6,7] owing to non-intuitive and unstable control caused by signal artifacts [8,9]. Multi-grip prosthetic hands have been recently made clinically available through the UK National Health Service [10] with the aim to improve function and overall user adherence. To facilitate this, advanced prosthetic hands comprising a greater number of sEMG electrodes have emerged. For instance, MyoPlus (Ottobock, Duderstadt, Germany) [11] and the COAPT system (COAPT LLC., Chicago, IL, USA) [12] utilize up to 17 sEMG electrodes to achieve 14 grip patterns. However, compared with commonly prescribed two-electrode myoelectric hands, multi-electrode hands are generally heavier and could also present increased risks associated with skin irritation [13]. Moreover, analysis of large-scale datasets has been reported [14] to limit efficiency of data processing and real-time feedback for prosthetic control. For the foreseeable future, global healthcare systems, particularly those in low-resource environments, may still prefer conventional low-cost myoelectric prosthetic hands, such as those based on two sEMG electrodes.

Machine learning has been identified [15] as a promising means to classify hand actions for more intuitive and accurate control of prosthetic hands. This typically entails feature extraction from raw sEMG signals, such as maximum, minimum, and standard deviation (SD) values obtained during muscle contraction. Extracted features are used to train machine learning models as a means to establish correspondence between sEMG signal features and hand actions, known as classification. Trained classification models can subsequently be used to classify hand actions based on new sEMG datasets, whereby classification accuracies can be calculated by dividing the number of correct predictions by the total number of predictions. Li et al. [16] reported classification accuracy of approximately 93% using linear discriminant analysis (LDA), artificial neural network, and fuzzy logic algorithms to categorize six hand actions with ten electrodes. This accuracy decreased to approximately 92% and 89% when reducing electrode count to six and four, respectively [16]. Using support vector machines (SVM) and k-nearest neighbors (KNN) algorithms, Mukhopadhyay et al. [17] reported accuracies of approximately 96% when categorizing eight hand actions using seven electrodes. An accuracy range of 79–82% was also reported [18] using LDA for ten hand actions with four electrodes. For multi-electrode systems, various feature types and different approaches to extract sEMG signal features have also been reported. For example, sEMG feature evaluation has been conducted [19] to improve pattern recognition robustness, which suggested that, among the fifty features extracted in the study, only four features were major contributors to achieve 99% accuracy when classifying four hand actions with four electrodes on a single subject. Moreover, a two-dimensional matrix image-based approach was reported [20] for classification of sEMG signals, which was compared using common machine learning algorithms, including KNN and SVM.

Despite their high accuracies, the aforementioned models are based on multi-electrode systems. For two-sEMG-electrode systems, machine learning algorithms and feature extraction have been reported [21] with a view to achieving similar classification accuracies. For example, robust hand gesture recognition based on two-electrode sEMG systems and SVM algorithms has been reported, where overall classification accuracies of over 90% [22] and 97% [23] were achieved. However, a recent review [24] reporting the use of sEMG signals to classify hand gestures highlighted the lack of focus on two-sEMG-electrode systems in the field. Additionally, classification accuracy based on two-sEMG-electrode systems was reported [25] to vary by up to 20%, depending on machine learning algorithms utilized. Thus, it is important to compare different classification algorithms and feature types to facilitate comprehensive machine learning evaluation. To the best of our knowledge, there is currently a lack of such comparative studies on machine learning algorithms based on signals produced by two-sEMG-electrode systems, which is the focus and main contribution of this work.

## 2. Materials and Method

**Figure 1:**
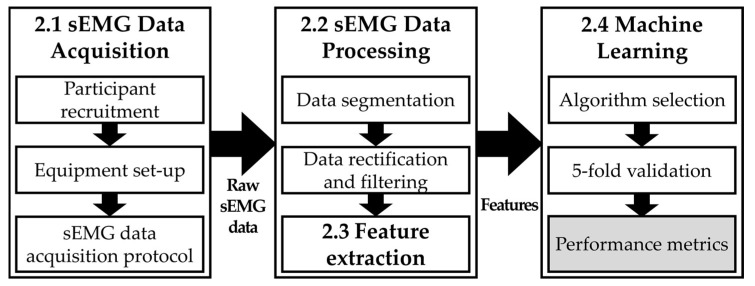
Study overview.

Figure 1 shows an overview of this study. In particular, during the initial sEMG data acquisition stage, two sEMG electrodes from a Delsys Trigno Research+ acquisition system were attached to the belly of the flexor and extensor carpi radialis muscles located in the forearm of an able-bodied participant. Each participant was instructed to conduct six designated hand actions while real-time sEMG signals were collected. Raw sEMG data collated from the nine participants were subsequently processed, including data segmentation, rectification, filtering, and signal feature extraction. The extracted features were then used with KNN, SVM, and LDA machine learning algorithms, respectively, to train classification models. Hand action classification accuracies were evaluated by varying the algorithm and number of features. Further details of each stage are included in the following sections, as also indicated in Figure 1.

### 2.1. sEMG Data Acquisition

**Figure 2:**
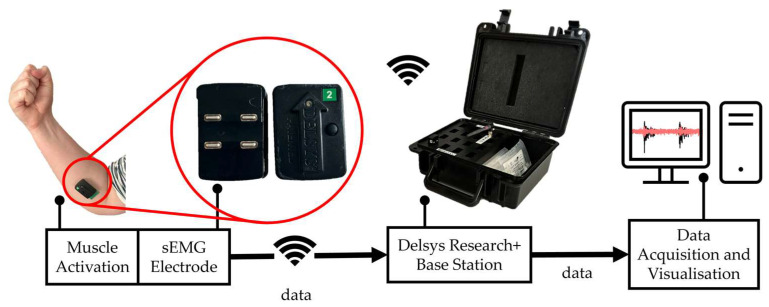
Block diagram of the measurement system illustrating steps involved in sEMG signal acquisition.

**Figure 3:**
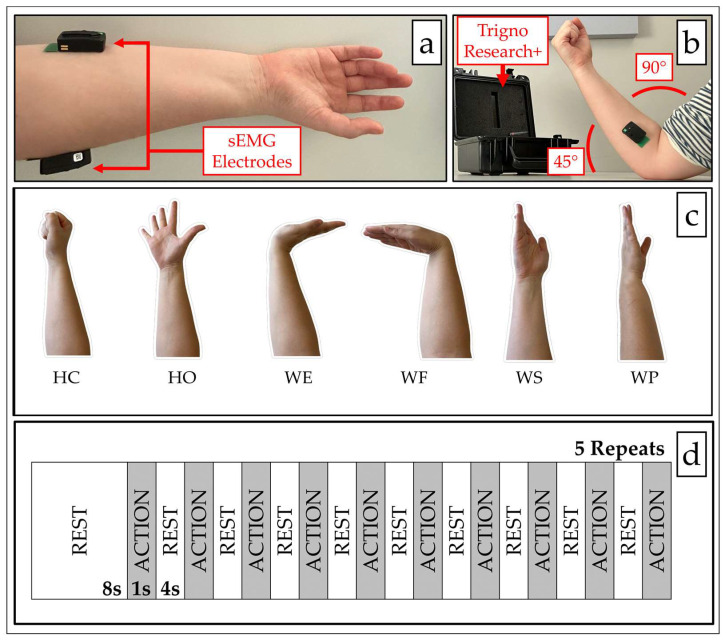
(**a**) sEMG electrodes placed on lateral and medial sides of a participant’s forearm. (**b**) A photo demonstrating hand and arm positions on a desk prior to data collection. (**c**) Illustrations of the six hand actions. (**d**) Time sequence of a hand action session.

Nine able-bodied participants without upper limb loss or injuries were recruited for this study (6 female, 3 male; 24 ± 6 years). This study utilized a Delsys Trigno Research+ acquisition system which included a base station, two wireless differential sEMG electrodes with a sampling frequency of 1111 Hz, and EMGWorks software (version 4.7.9, Delsys Inc., Natick, MA, USA), illustrated in Figure 2 [26]. Each participant was instructed to perform wrist flexion and extension, enabling the researcher to feel for the approximate locations of the flexor carpi radialis and extensor carpi radialis. Subsequently, two sEMG electrodes were attached to the right forearm at the belly of the flexor carpi radialis (medial electrode) and the extensor carpi radialis (lateral electrode) using double-sided adhesive, as shown in Figure 3a. Electrode placement protocol was based on Surface ElectroMyoGraphy for the Non-Invasive Assessment of Muscles (SENIAM) recommendations [27], which describes optimal sEMG sensor application procedures. Electrode locations were selected in line with previous studies [28,29,30].

Following the electrode setup, each participant was instructed to sit and rest their forearms on a desk at an angle of approximately 45° to create a 90° elbow angle (Figure 3b). Subsequently, each participant was asked to conduct six hand actions, including hand open (HO), hand close (HC), wrist extension (WE), wrist flexion (WF), wrist pronation (WP), and wrist supernation (WS), as shown in Figure 3c. These hand actions represent agonist and antagonist movements that trigger flexor and extensor muscle groups within the forearm, and thus are commonly exploited for sEMG signal processing in myoelectric control research [31,32,33].

A session was defined as one data recording consisting of 10 repetitions of the same hand action, as shown in Figure 3d. Each session commenced with the participant resting their hand for approximately eight seconds. A hand action was initiated and held for one second, which was then repeated ten times. The process was cued by a visual prompt, with a hand rest period lasting for four seconds in between. A time sequence of a typical session is shown in Figure 3d. Each hand action session was repeated five times. In total, each hand action was performed 50 times for each participant.

### 2.2. sEMG Data Processing

**Figure 4:**
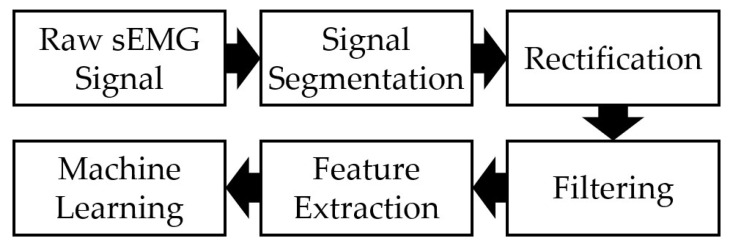
Signal processing block diagram.

**Figure 5:**
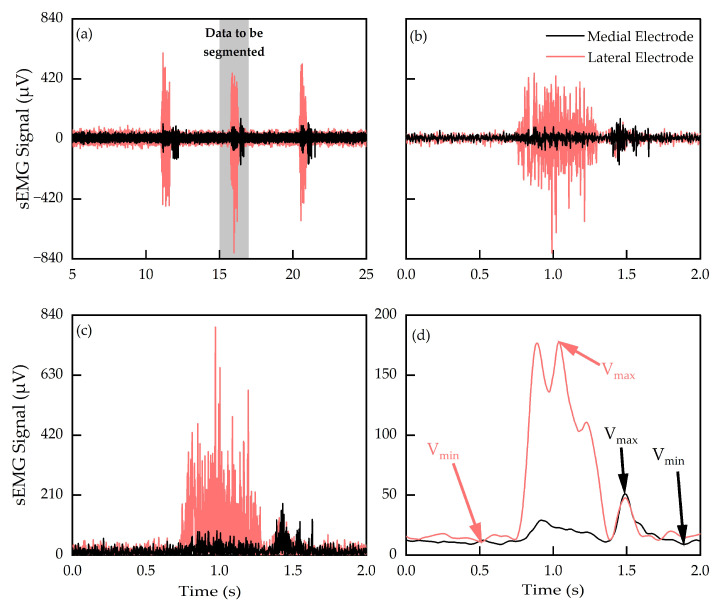
(**a**) Typical sEMG signals from WE action as a function of time, (**b**) segmented signals, (**c**) full-wave rectified signals, and (**d**) filtered signals, including exemplar identification of features of minimum and maximum voltages (V_min_ and V_max_).

Figure 4 outlines the data processing protocol. Figure 5a shows typical sEMG signals generated during WE actions, and Figure 5b shows the corresponding segmented signals. Signal segments were defined based on the time sequence, as shown in Figure 3d. The duration of each signal segment was two seconds, ensuring the signal associated with a one-second hand action was fully encased. The start and end points of each segment were positioned so that the location of peak signal activity across both electrodes occurred at the center of that segment. Full-wave rectification [34] was then performed on the segmented signals (Figure 5c), converting negative signal components into positive counterparts. Subsequently, a third-order low-pass Butterworth Filter with a cut-off frequency of 5 Hz was applied to the rectified signals (Figure 5d), producing envelope-like profiles. The filtered signals were subsequently used for feature extraction. Pearson’s correlation coefficient was calculated using filtered sEMG signals from both electrodes to study the correlation between the sEMG signals produced by the medial and lateral electrodes. Low correlation was defined as a correlation coefficient of 0.5 or below [35].

### 2.3. Feature Extraction

Six features were extracted from full-wave rectified and filtered sEMG signals for each electrode. This resulted in a total of 12 features per data segment, including mean voltage (V_mean_), maximum voltage (V_max_), minimum voltage (V_min_), standard deviation of voltage (V_SD_), skewness (skew) of voltage, and kurtosis (kurt) of voltage. These features have been previously adopted for pattern recognition using sEMG signal classification [36]. Table 1 summarizes equations that were used to determine feature values of V_max_, V_min_, V_mean_, V_SD_, skewness, and kurtosis. These features were subsequently input into machine learning models without calibration or standardization to simplify the process.

### 2.4. Machine Learning

**Figure 6:**
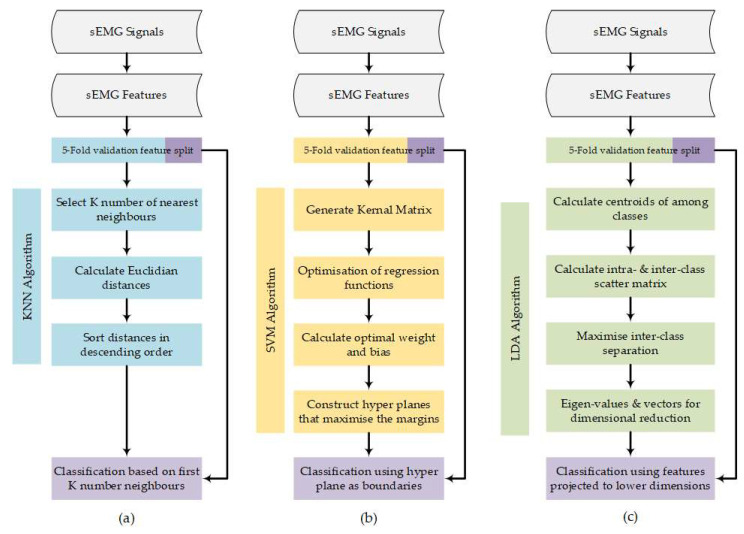
Flowchart outlining machine learning algorithms for (**a**) KNN, (**b**) SVM, and (**c**) LDA.

Features obtained from each participant were used to train KNN, LDA, and SVM algorithms, respectively. The flowchart in Figure 6 outlines the underlying principles for KNN (Figure 6a) [37], SVM (Figure 6b) [38], and LDA (Figure 6c) [39] algorithms. Overall classification accuracy, along with action-specific accuracy, precision, sensitivity, and F1-score [40] were used to define a model’s performance metrics. Model performance metrics were subsequently evaluated with respect to the number of features used (up to 12) for training. All mean performance metrics were calculated using all possible combinations of the 12 features across the two electrodes.

A 5-fold validation process [41] was used to quantify classification performance for each machine learning model. Confusion matrices were subsequently generated using feature values, enabling calculation of four parameters for each hand action. These are true positive (TP), true negative (TN), false positive (FP), and false negative (FN).

Action-specific performance metrics, displayed in Equations (7)–(10), were calculated.

(7) $$\mathrm{Action}\;\mathrm{Precision}=\frac{\mathrm{TP}}{\mathrm{TP}+\mathrm{FP}}$$

(8) $$\;\mathrm{Action}\;\mathrm{Sensitivity}\;=\;\frac{\mathrm{TP}\;}{\mathrm{TP}\;+\;\mathrm{FN}}$$

(9) $$\mathrm{Action-Specific}\;\mathrm{Accuracy}\;=\;\frac{\mathrm{TP}\;+\;\mathrm{TN}}{\mathrm{TP}+\mathrm{FP}+\mathrm{TN}+\mathrm{FN}}$$

(10) $$\mathrm{Action}\;\mathrm{F1-Score}\;=\;\frac{2\;\times \;\mathrm{Precision}\;\times \;\mathrm{Sensitivity}}{\mathrm{Precision}+\mathrm{Sensitivity}}$$

Overall classification accuracy across all hand actions can be defined as the percentage of total TP, shown in Equation (11).

(11) $$\mathrm{Overall}\;\mathrm{Classification}\;\mathrm{Accuracy}\;=\;\frac{\sum TP}{\mathrm{Total}\;\mathrm{Data}\;\mathrm{Samples}}\;$$

## 3. Results

### 3.1. Processed sEMG Signals

**Figure 7:**
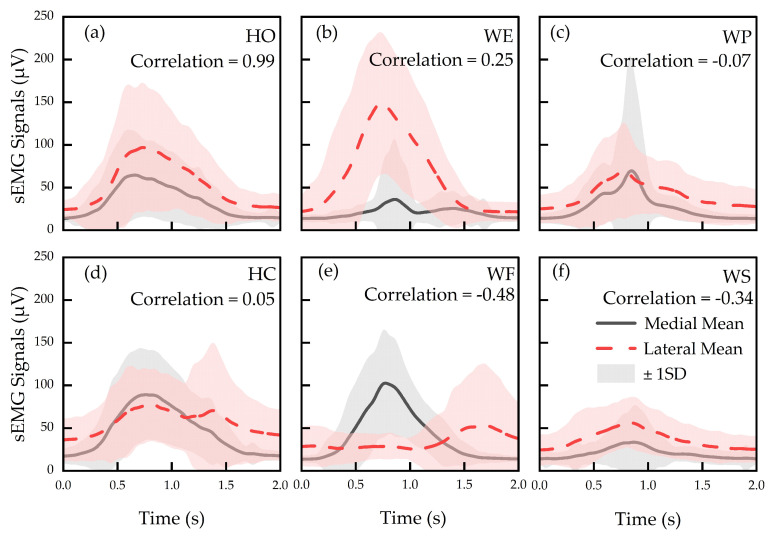
Mean and ±1 SD of sEMG signals for (**a**) HO, (**b**) WE, (**c**) WP, (**d**) HC, (**e**) WF, and (**f**) WS actions.

Figure 7 shows mean and ±1 SD of processed sEMG signals obtained from all participants, plotted on a range from 0 to 250 μV. Considering all hand actions, HO produced the highest correlation coefficient of 0.99 (Figure 7a), while HC resulted in the lowest correlation coefficient of 0.05 (Figure 7d). WE, WF, WS, and WP produced correlations of 0.25, −0.48, −0.34, and −0.07, respectively.

Table 2 summarizes the mean of feature values across all participants, which ranged within 18.6–53.8 µV (V_mean_), 62.5–204.5 µV (V_max_), 9.0–23.2 µV (V_min_), and 10.6–52.1 µV (V_SD_). Skewness and kurtosis fell in the ranges of 1.5–2.4 and 5.1–10.2, respectively. There is a distinct difference between V_max_ values from the medial and lateral electrodes for WE and WF. For instance, WE led to V_max_ of 81.8 µV and 204.5 µV across the medial and lateral electrodes, respectively, while WF led to V_max_ of 171.0 µV and 102.3 µV across the medial and lateral electrodes. This may be due to the fact that electrodes were placed directly on the corresponding activation muscles, namely extensor carpi radialis and flexor carpi radialis during WE and WF. On the other hand, V_max_ values obtained from medial and lateral electrodes for HC were 155.5 µV and 153.2 µV, respectively, while V_max_ values obtained from medial and lateral electrodes during HO were 101.4 µV and 148.4 µV, respectively. The difference in V_max_ between the two electrodes during HC and HO was minimal. This may be because HO and HC actions require activations from similar muscle groups and thus the V_max_ feature may not be best suited to distinguish these types of hand actions. However, it was noted that the medial electrode resulted in V_SD_ of 22.5 for HO and 34.9 for HC, which could be used as dominant features in the classification algorithm. Nonetheless, this study included all features to train machine learning models with a view to focus on comparing the machine learning algorithms.

### 3.2. Action-Specific Performance Metrics

**Figure 8:**
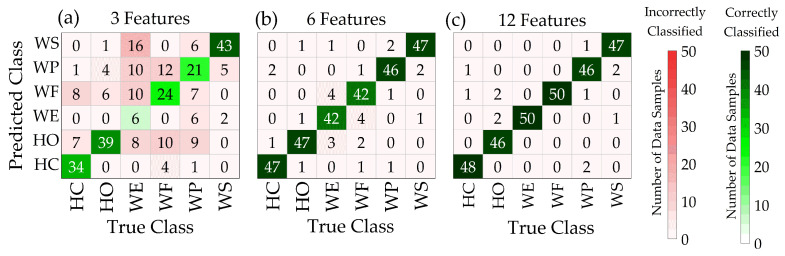
Example of confusion matrices obtained with SVM algorithm based on signals obtained from one participant using (**a**–**c**).

Figure 8 displays typical examples of confusion matrices generated from one participant using SVM. It is worth noting that features used for classification were randomly selected for the three-feature and six-feature models.

For the three-feature model, WE action was correctly classified six times (third row and third column in Figure 8a) and incorrectly classified 44 times, resulting in a classification accuracy of 12%. This increased to 42 times (Figure 8b) and 50 times (Figure 8c) when the number of features increased to six and twelve, respectively. TP, TN, FP, and FN were calculated using confusion matrices generated from the machine learning models using all possible feature combinations. Subsequently, performance metrics (using Equations (7)–(11)) were calculated using all possible feature combinations.

**Figure 9:**
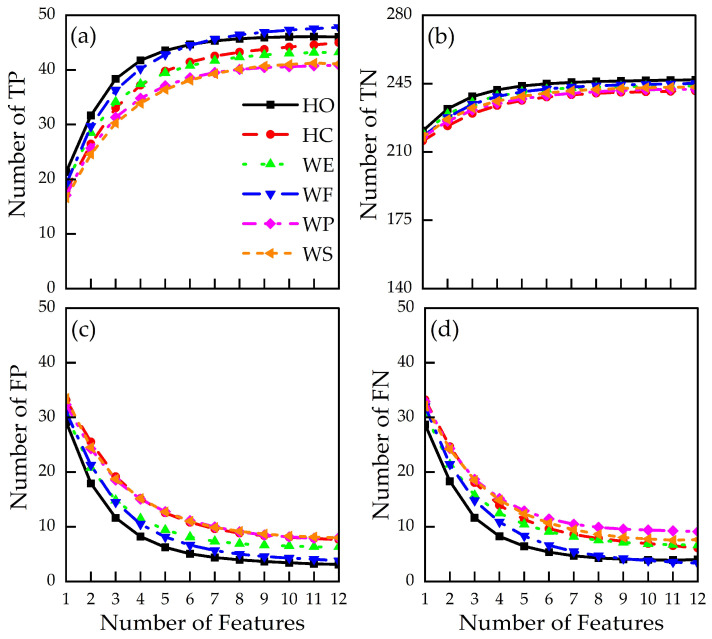
Mean profiles of (**a**) true positive (TP), (**b**) true negative (TN), (**c**) false positive (FP), and (**d**) false negative (FN) across all participants.

**Figure 10:**
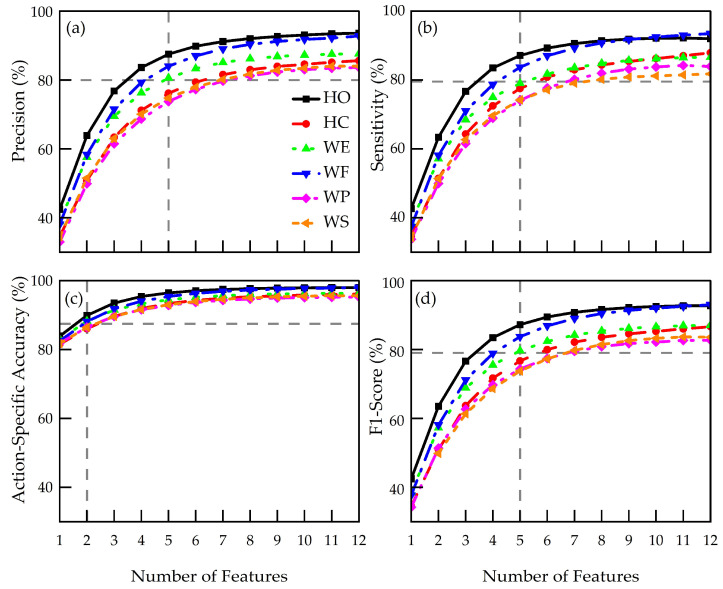
Mean profiles of (**a**) precision, (**b**) sensitivity, (**c**) action-specific accuracy, and (**d**) F1-score across all participants. Vertical lines indicate plateau onset values for number of features and horizontal lines indicate corresponding mean performance metrics.

Figure 9 displays mean TP, TN, FP, and FN values as a function of the number of features for the six different hand actions. WF action resulted in the highest number of correct classifications of all hand actions (maximum TP of 47.8), whereas WP and WS show the lowest number of correct classifications (maximum TP of 40.9 and 41.1, respectively). HO and WF were shown to have the lowest false classification, namely FP (Figure 9c) and FN (Figure 9d), dropping below 5 (10% of total data samples). TN (Figure 9b) has a mean maximum value of 243.7, further demonstrating a high level of correct classification. Performance metrics, as shown in Figure 10, were subsequently calculated using TN, TP, FP, and FN values based on Equations (7)–(11).

Figure 10 displays mean values of action-specific performance metrics as a function of number of features. It is evident that all performance metrics increased with number of features and eventually plateaued. We defined the onset of the plateau as the point at which relative change between consecutive feature accuracies was within 5%. For precision (Figure 10a) and sensitivity (Figure 10b), profiles began plateauing at approximately five features, with mean corresponding accuracies of 79 ± 8% and 79 ± 8%, respectively, with both reaching a maximum accuracy of 88 ± 6%. Action-specific accuracy (Figure 10c) began to plateau at two features (corresponding to a mean of 88 ± 2%), reaching a maximum of 97 ± 2%. F1-score (Figure 10d) plateaued at five features, corresponding to a mean value of 79 ± 8%, and reached a maximum value of 87 ± 7%.

### 3.3. Overall Accuracy

**Figure 11:**
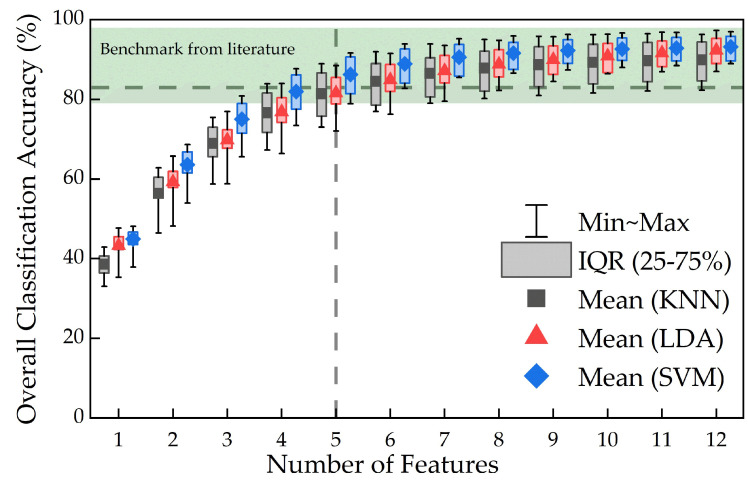
Overall classification accuracy as a function of number of features averaged across all participants, where the corresponding values in the literature are indicated by the shaded band and the dashed line indicates accuracy at which plateauing occurs.

Figure 11 shows overall classification accuracy for each algorithm in relation to number of features using all possible feature combinations. Overall accuracy in the range of 30–95% was achieved for the selected numbers of features, with a maximum overall classification accuracy of 93 ± 2%. Akin to Figure 10, a logarithmic relationship was also revealed, which started to plateau at five features (83%). Overall classification accuracy achieved using SVM algorithm was slightly higher than those from KNN and LDA algorithms. It should be noted that the minimum–maximum range is relatively low (13 ± 5%) for all data points, representing low variation across participants.

**Figure 12:**
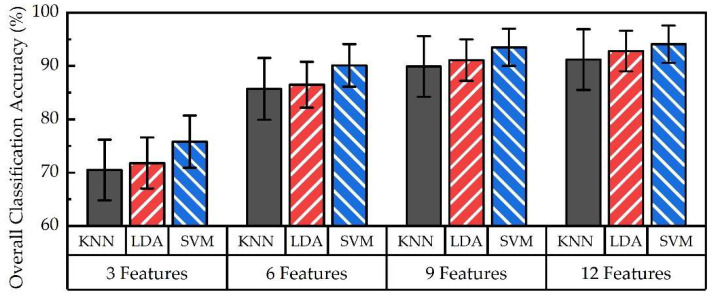
Comparison of overall classification accuracy across KNN, LDA, and SVM algorithms for 3, 6, 9, and 12 features.

Figure 12 compares overall classification accuracy across KNN, LDA, and SVM classification algorithms. It is evident that SVM resulted in higher accuracy (up to 93 ± 3%) than that of LDA (up to 92 ± 4%) and KNN (up to 90 ± 5%). This trend is consistent regardless of the number of features used for classification.

## 4. Discussion

This study utilized sEMG signals from two electrodes to classify typical hand actions. A high correlation coefficient from HO actions (0.99) is shown in Figure 7a. This may be attributable to activation of dorsal interossei muscles, located between the fingers during HO action. However, corresponding sEMG signals from medial and lateral forearm muscles likely represent subsequent movement associated with wrist stabilization, which requires concurrent contractions, and thus can be considered complementary. In contrast, HC actions (Figure 7d) resulted in the lowest correlation coefficient (0.05). This may potentially be due to contractions of the palmaris longus (medial area of the forearm) required during HC action, whereas the lateral muscles play a secondary and assistive role to ensure wrist stabilization for different actions. Low correlation coefficients (−0.5 to 0.5) were obtained for all other hand actions, which indicates limited crosstalk between medial and lateral sEMG signals. This was expected because the two electrodes were placed at opposite sides of the forearm on two distinct muscle groups. Crosstalk reduction among electrodes is desirable for signal processing since multi-collinearity caused by crosstalk may lead to unstable numerical solutions and model bias [42], which are known factors affecting classification accuracy. It is plausible to note that high-density sEMG multi-electrode systems (i.e., >2 electrodes) may incur greater signal crosstalk than those of two-sEMG-electrode systems. Thus, a tradeoff between advanced functions and accuracies may need to be considered in future multi-electrode systems.

Precision (88 ± 6%, in Figure 10a) and sensitivity (88 ± 6%, Figure 10b) were lower than previously reported values, i.e., 98% [43,44]. Considering the function-driven nature of a prosthetic hand, precision is usually considered more important than sensitivity for classification models [45]. Imprecise classification models may result in a greater number of false positives, which may subsequently lead to unstable control of a prosthesis. Real-world consequences include unprovoked prosthetic hand activations, resulting in items being dropped or gripped harder than intended, both of which have been frequently reported in this field [46] as one of the main contributing factors for high rejection rates [9]. On the other hand, low sensitivity could be associated with higher rates of false negatives, for instance, when a prosthesis user intends to perform a hand action, but the activation is not realized. Such devices could require users to overexert themselves when producing muscle activations, potentially leading to muscle fatigue or damage over time [47]. Consequently, such prosthetic hands are often deemed less responsive, which has also been reported to potentially be associated with device rejection [48].

Figure 10 and Figure 11 indicate logarithmic relationships between the number of features used and corresponding performance metrics. Classification accuracy plateaued at five features, indicating a small difference in classification performance (i.e., relative difference of less than 5%) when further increasing the number of features beyond five. However, this logarithmic relationship may be dependent on the type and number of features selected. While we only used time-domain features in this study, features in the frequency or time-frequency domain, for instance, may further increase classification accuracy. Additionally, multimodal sensor fusion via addition of other sensing features, such as inertial measurement units for motion detection [49] and/or force sensors for fingertip haptic feedback [50], could also be employed to create a more data-rich environment in order to improve overall accuracy. In those cases, it would be prudent to consider the potential constraints on data transfer and processing for large-scale datasets. Nonetheless, these results suggest there may be a limit to the number of features required to achieve high performance metrics when using two-sEMG-electrode prosthetic systems.

Figure 10 shows high action-specific classification accuracies (>90%) and F1-scores (>80%) were achieved for each hand action. High F1-scores obtained in this study suggest that in such a two-sEMG-electrode setup, features and machine learning algorithms adopted may be suitable for future prosthetic hand controls and actuation. Figure 11 shows high overall classification accuracies (93 ± 2%) can be achieved using sEMG signals from only two electrodes, which falls in the range of those reported in previous studies (79–99%) [16,17,18,19,20,22,23]. In addition, a relatively small variation in overall classification accuracy (13 ± 5%) indicates accuracy is somewhat not dependent on participants’ capabilities. We thus believe there could still be research potential in this field, particularly with a view of unravelling and optimizing the performance of these two-electrode systems. This could be performed in parallel with the development of multi-electrode myoelectric prosthetic hands, thus presenting alternative options to meet users’ multi-faceted needs.

In this study, highest accuracy was achieved by SVM (up to 93 ± 3%) when classifying six hand actions, as compared with LDA (up to 92 ± 4%) and KNN (up to 90 ± 5%), as shown in Figure 12. This may be due to the fact that SVM algorithm training protocol is based on maximizing the separation of classification boundaries for different hand actions. Separation of classification boundaries is primarily sensitive to sEMG features generated by distinctive hand actions, such as WF and WE. Feature values located at a greater distance from the classification boundary would not notably affect classification performance. In contrast, should a hand action, such as HO and HC, involve sEMG signals generated by similar muscle groups, the separation of classification boundaries would reduce, which may affect overall accuracy. For those types of hand actions, using an alternative algorithm or hybrid algorithms may be favorable compared to SVM. It is worth noting that detailed comparisons across different machine learning algorithms for hand action classification have not been performed. Comprehensive machine learning evaluation may be advisable to inform appropriate selection of classification algorithms best suited for desired hand actions. This may further inform potential design of upper limb prostheses incorporating machine learning.

Results produced in this study suggest SVM may be well-suited for classifying hand actions with distinct muscle contractions, such as WE and WF actions. However, use of hybrid machine learning models may potentially affect the separation of classification boundaries for different hand actions with similar muscle activation patterns, which could be explored in future work. In particular, SVM algorithm could be explored when used in conjunction with sine cosine and cuckoo search algorithms [51], which may facilitate studies involving a wider variety of hand actions. Although use of advanced algorithms may notably increase computational demand, they may potentially enable improved control of myoelectric prosthetic hands.

Future work could incorporate feature calibration to include scaling and normalization, which may further improve classification accuracy. Additionally, this study focused on the mean performance metrics at a set number of features. Further investigation could be conducted to better understand the impact of specific features, which may help to achieve optimized classification models. While this study involved comparisons of three commonly used machine learning algorithms, other algorithms such as logistic regression, Naive Bayes, and decision tree, as well as hybrid algorithms could also be explored with a view to further improve overall classification accuracies. State-of-the-art hybrid machine learning algorithms could be adapted to decipher and classify real-time sEMG signals with additional hand actions. Examples of these include two-phase cuckoo search-based deep learning [51] or deep learning models combining auto-encoders and neural networks [43].

## 5. Conclusions

This study reports the acquisition, processing, and machine learning analysis of two- sEMG-electrode signals obtained using a Delsys Trigno Research+ acquisition system. sEMG signals for six common hand actions were attained from nine able-bodied participants. Twelve sEMG features were extracted for machine learning studies to facilitate comparison of KNN, SVM, and LDA algorithms. Hand action classification accuracies were evaluated and compared across these algorithms. Classification accuracy was also studied as a function of the number of extracted features. Results revealed that overall classification accuracy of 93 ± 2% can be achieved based on two-electrode sEMG signals, which is in line with values reported in the literature. Individual hand action classification accuracy was found to reach 97 ± 2%, while action precision and sensitivity of up to 88 ± 6% were obtained. A logarithmic relationship between number of selected features and classification accuracy was found, which also indicated five features could be sufficient to achieve a reasonably high overall accuracy of 83%. Moreover, the effect of machine learning algorithms on overall classification accuracy was demonstrated, with the highest overall accuracy achieved using SVM (93 ± 3%), followed by LDA (92 ± 4%) and KNN (90 ± 5%). Our analysis suggests that it may be advisable to evaluate suitable classification algorithms based on different hand action selections. Future work could include further studies involving other machine learning classification algorithms, such as hybrid machine learning models, which may potentially reduce the separation of classification boundaries for hand actions with similar muscle activation patterns. Inclusion of participants with upper limb loss could also enable study of physiological differences in muscle actions.

## Figures and Tables

**Table 1:** List of the features with corresponding equations and definitions.

Feature	Equation		Definition
V_mean_	$\frac{1}{n}\sum_{i=1}^{n}V_{i}$	(1)	The mean absolute voltage.
V_max_	$max(V_{i})$	(2)	The maximum absolute voltage value.
V_min_	$min(V_{i})$	(3)	The minimum absolute voltage value.
V_SD_	$\sqrt{\frac{1}{n-1}\sum_{i=1}^{n}{\left|V_{i}-V_{\mathrm{mean}}\right|}^{2}}$	(4)	The standard deviation of the absolute voltage.
Skew	$\frac{\sum_{i}^{n}{\left(V_{i}-V_{\mathrm{mean}}\right)}^{3}}{{\left(\sqrt{\frac{1}{n}\sum_{i=1}^{n}{\left(V_{i}-V_{\mathrm{mean}}\right)}^{2}}\right)}^{3}}$	(5)	A measure of asymmetry.
Kurt	$\;\frac{\frac{1}{n}\sum_{i=1}^{n}{\left(V_{i}-V_{\mathrm{mean}}\right)}^{4}}{{\left(\frac{1}{n}\sum_{i+1}^{n}{\left(V_{i}-V_{\mathrm{mean}}\right)}^{2}\right)}^{2}}$	(6)	A measure of tailedness.
n: total number of sEMG signal samples; V_i_: ith sEMG signal sample after rectification and filtering			

**Table 2:** Mean of feature values obtained from all participants.

**Medial**						
**Action**	**V_mean_ (µV)**	**V_max_ (µV)**	**V_min_ (µV)**	**V_SD_ (µV)**	**Skew**	**Kurt**
HO	28.5	101.4	10.6	22.5	1.8	6.2
HC	38.7	155.5	11.7	34.9	1.8	6.5
WF	33.8	171.0	9.7	36.8	2.1	7.0
WE	19.0	81.8	9.0	12.7	2.4	10.2
WS	18.6	62.5	10.5	10.6	2.1	8.3
WP	23.8	121.6	9.2	21.7	2.0	7.9
**Lateral**						
**Action**	**V_mean_ (µV)**	**V_max_ (µV)**	**V_min_ (µV)**	**V_SD_ (µV)**	**Skew**	**Kurt**
HO	45.8	148.4	16.1	33.1	1.5	5.2
HC	51.9	153.2	23.2	28.6	1.6	5.7
WF	33.6	102.3	14.6	18.2	1.9	7.7
WE	53.8	204.5	13.8	52.1	1.6	5.1
WS	33.4	83.0	17.1	15.0	1.5	5.6
WP	37.2	109.4	17.2	19.6	1.6	6.3

## Data Availability

All data supporting this study are openly available from the University of Southampton repository at https://doi.org/10.5258/SOTON/D2941 (accessed on 24 January 2024).
